# A liposomal carbohydrate vaccine, adjuvanted with an NKT cell agonist, induces rapid and enhanced immune responses and antibody class switching

**DOI:** 10.1186/s12951-023-01927-x

**Published:** 2023-06-02

**Authors:** Ji-Xiang Jia, Sen-Lin Peng, Ndayambaje Yvan Kalisa, Qiang Chao, Zhifang Zhou, Xiao-Dong Gao, Ning Wang

**Affiliations:** 1 Key Laboratory of Carbohydrate Chemistry and Biotechnology, Ministry of Education, School of Biotechnology, Jiangnan University, Wuxi, 214122 China; 2 State Key Laboratory of Biochemical Engineering, Institute of Process Engineering, Chinese Academy of Sciences, Beijing, China

**Keywords:** Carbohydrate vaccine, Liposome nanoparticles, Adjuvant, Congenital disorders of glycosylation, Antibody class switching

## Abstract

**Background:**

Congenital disorders of glycosylation (CDGs) are genetic diseases caused by gene defects in glycan biosynthesis pathways, and there is an increasing number of patients diagnosed with CDGs. Because CDGs show many different clinical symptoms, their accurate clinical diagnosis is challenging. Recently, we have shown that liposome nanoparticles bearing the ALG1-CDG and PMM2-CDG biomarkers (a tetrasaccharide: Neu5Ac-α2,6-Gal-β1,4-GlcNAc-β1,4-GlcNAc) stimulate a moderate immune response, while the generated antibodies show relatively weak affinity maturation. Thus, mature antibodies with class switching to IgG are desired to develop high-affinity antibodies that may be applied in medical applications.

**Results:**

In the present study, a liposome-based vaccine platform carrying a chemoenzymatic synthesized phytanyl-linked tetrasaccharide biomarker was optimized. The liposome nanoparticles were constructed by dioleoylphosphatidylcholine (DOPC) to improve the stability and immunogenicity of the vaccine, and adjuvanted with the NKT cell agonist PBS57 to generate high level of IgG antibodies. The results indicated that the reformulated liposomal vaccine stimulated a stronger immune response, and PBS57 successfully induce an antibody class switch to IgG. Further analyses of IgG antibodies elicited by liposome vaccines suggested their specific binding to tetrasaccharide biomarkers, which were mainly IgG2b isotypes.

**Conclusions:**

Immunization with a liposome vaccine carrying a carbohydrate antigen and PBS57 stimulates high titers of CDG biomarker-specific IgG antibodies, thereby showing great potential as a platform to develop rapid diagnostic methods for ALG1-CDG and PMM2-CDG.

**Graphical Abstract:**

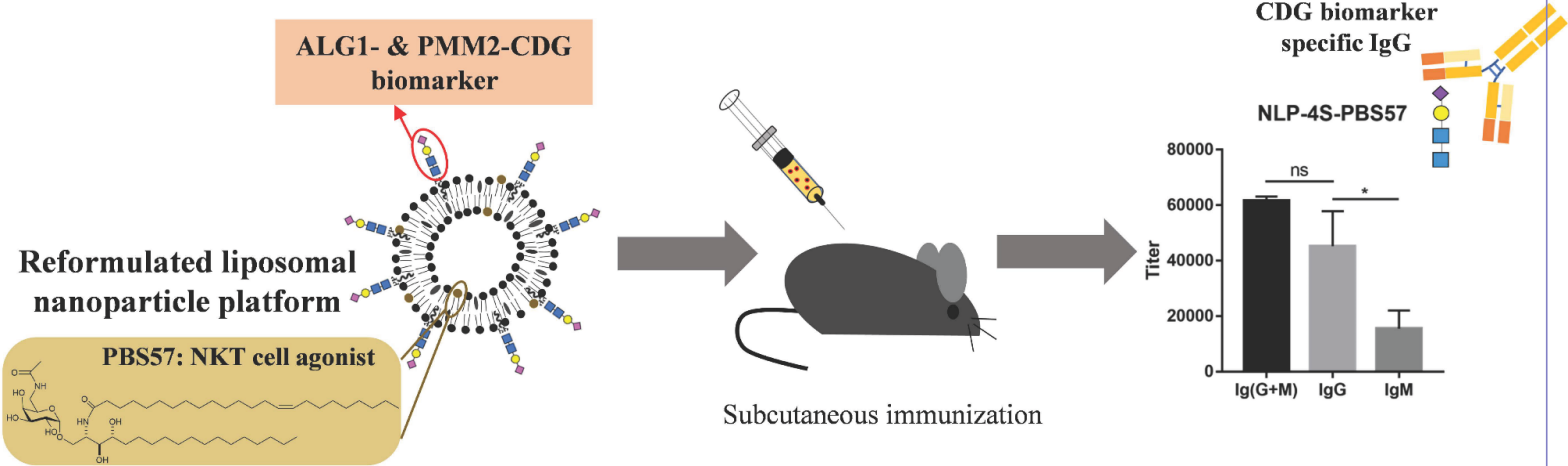

## Introduction

Congenital disorders of glycosylation (CDGs) are genetic diseases caused by defects in genes that encode related enzymes in the oligosaccharide biosynthesis pathways [1, 2]. The eukaryotic N-glycan biosynthesis pathway starts from the production of lipid-linked oligosaccharide (LLO) precursors in the endoplasmic reticulum (ER) [3, 4]. During this process, β1,4-mannosyltransferase (ALG1) and phosphomannomutase-2 (PMM2), which act in the early stage, play critical roles [5–8]. In humans, defects in *ALG1* or *PMM2* lead to poorly functioning enzymes, which further result in aberrant glycoproteins in ALG1-CDG and PMM2-CDG, respectively [9, 10]. As the most and third most common type of CDG, the numbers of patients suffering from PMM2-CDG (~ 1000 cases) and ALG1-CDG (~ 100 cases), respectively, are rapidly increasing worldwide [11–13]. CDGs usually present with variable clinical features that affect nearly all systems, such as psychomotor retardation and dysmorphia, which cause clinical difficulties in diagnosis [13, 14]. Consequently, a fast and specific diagnostic method for the precise identification of certain types of CDGs is needed.

**Fig. 1:**
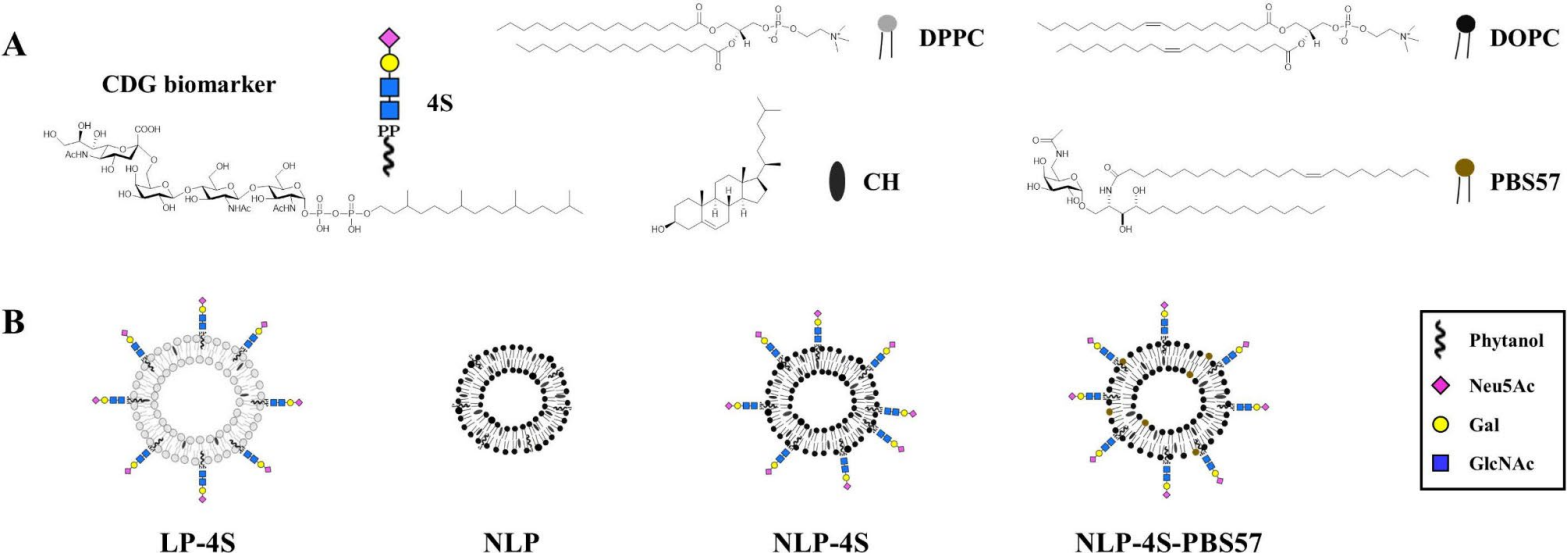
Composition and structure of liposome vaccines. **A** Chemical structures of 4S, DPPC, CH, DOPC and PBS57. 4S is a lipid-modified CDG biomarker that can be loaded onto liposome membranes. DPPC, DOPC and CH are the main components of liposomes. Adjuvant PBS57 is added to enhance the switching of the antibody class from IgM to IgG. **B** Schematic structures of **LP-4S**, **NLP**, **NLP-4S** and **NLP-4S-PBS57** liposome vaccines. **LP-4S** is prepared by DPPC, CH and loaded with CDG biomarker antigen 4S. **NLP** is prepared by DOPC and CH as the blank liposome. **NLP-4S** is prepared by DOPC, CH and loaded with CDG biomarker antigen 4S. **NLP-4S-PBS57** is prepared by further loading PBS57 adjuvant into **NLP-4S**.

Currently, CDG patients can be successfully screened by gene sequencing [15, 16]. Other diagnostic methods include MS-based serum/plasma N-glycomics and serum transferrin pattern analysis, which are time-consuming and expensive but less specific to a certain defect gene [2, 17–19]. Recently, one tetrasaccharide, Neu5Ac-α2,6-Gal-β1,4-GlcNAc-β1,4-GlcNAc (SiaGalGlcNAc2), has been detected in the sera of ALG1- and PMM2-CDG patients as a biomarker and has great potential to be used in clinical studies [9, 20]. To develop the diagnostic method for CDGs, we previously reported the in vitro chemoenzymatic synthesis of SiaGalGlcNAc2-PP-Phy (4S, Fig. 1A), which was used as an antigen to explore whether specific antibodies can be elicited [21]. Utilizing its lipid tail, 4S was loaded onto a liposome-based vaccine platform constructed by dipalmitoylphosphatidylcholine (DPPC, Fig. 1A) and cholesterol (CH, Fig. 1A). Taking advantage that the structure and properties are similar to those of biological phospholipid bilayer membranes, DPPC liposomes usually show no toxicity and can maintain glycolipid antigen stability in vivo [22]. Unfortunately, although the DPPC liposomal vaccine (**LP-4S**, Fig. 1B) stimulated an immune response in mice to produce specific antibodies against ALG1- and PMM2-CDG biomarkers, the antibody titers were relatively low and high levels of IgM rather than IgG [21], which restricted its use as a diagnostic tool.

Oligosaccharides usually induce relatively weak antibody responses and generate IgM on B cell surfaces [23, 24]. To trigger the production of high-affinity antibodies and long-term memory responses, presentation of multivalent carbohydrate epitopes and promotion of class switching from IgM to IgG are needed. Liposomal nanoparticles are capable of carrying multivalent glycolipid antigens; thus, the composition of liposomes is a design component to improve immunogenicity [25, 26]. In addition, some immune cells, such as activated T cells and NKT cells, interact with B cells and play essential roles in class switching and affinity maturation [27–29]. It is well known that NKT cells recognize lipid antigens presented by CD1d, which binds to the α-galactosylceramide (αGalCer) glycolipid ligand [30, 31]. Glycosylceramide PBS57 (Fig. 1A) is another highly potent NKT cell agonist, which has been demonstrated to provide T cell help to B cells for antibody class switching and stimulate cytokine release in vivo [32, 33]. In the present study, we first optimized the components of a liposomal vaccine to prepare newly formulated liposomes using dioleoylphosphatidylcholine (DOPC, Fig. 1A) and CH [34, 35]. Moreover, the NKT cell activator, PBS57, was added as the adjuvant to improve the production of IgG antibodies. These approaches to develop liposomal carbohydrate vaccines enhanced the immune response and generated high titers of specific IgG antibodies targeting the tetrasaccharide CDG biomarker, which are promising for developing rapid clinical diagnostic methods for ALG1-CDG and PMM2-CDG.

## Results

In the present study, the synthetic CDG biomarker with or without the NKT cell agonist, PBS57, was loaded into bilayer neutral liposomes (Fig. 1B). The physical properties of the resulting liposomes were characterized, and the liposomal vaccines were tested for their ability to elicit the immune response in mice.

## Synthesis of antigen 4S and preparation of liposomes

The ALG1- and PMM2-CDG biomarker-containing nanoparticles are composed of antigen tetrasaccharide and self-assembled liposomes, which comprise a versatile platform to bring multiple copies of active molecules together. SiaGalGlcNAc2-PP-Phy (4S, Fig. 1A) was synthesized by chemoenzymatic methods as previously reported [21]. Starting from the GlcNAc2-PP-Phy substrate, sequential addition of Gal and Sia residues was catalyzed by *h*GalT and *Pd*SiaT, generating 4S, whose lipid tail (phytanyl) inserted into the liposome membrane, exposing the tetrasaccharide antigen on the surface to maintain immune-stimulating activity. Rather than the liposome we previously used [21], which was composed of DPPC and CH (4:1, molar ratio), we constructed and evaluated a new neutral liposome platform consisting of DOPC and CH (2:1, molar ratio) in the present study (Fig. 1B). Liposomes bearing phytanyl phosphate (Phy-P), which shared the lipid tail structure of 4S, were prepared as the blank liposome (**NLP**). Liposomes were manufactured using a thin-film method [36, 37], rehydrated in HEPES buffer and sonicated before use. The compositions of each liposome are listed in Table 1.

**Table 1:** Formulation and characterization of liposomal vaccines

	Composition (molar ratio)	Antigen/Adjuvant	Size (nm)	PDI	Zeta potential (mV)
**LP-4S**	DPPC:CH = 4:1	4S	236.8 ± 0.46	0.361 ± 0.017	-21.8 ± 0.8
**NLP**	DOPC:CH = 2:1	Phy-P	99.3 ± 0.7	0.184 ± 0.023	-11.7 ± 1.55
**NLP-4S**	DOPC:CH = 2:1	4S	126.6 ± 1.1	0.157 ± 0.006	-19.2 ± 1.84
**NLP-4S-PBS57**	DOPC:CH = 2:1	4S + PBS57	152.9 ± 6.9	0.267 ± 0.014	-22.5 ± 1.97

Liposomal particles were formulated as described. Particle size distribution, zeta potential and PDI were measured three times with a DLS spectrophotometer. Data are expressed as the mean ± standard deviation.

## Characterization of liposomes

To characterize the above liposome formulations, the physical properties of all liposomes were quantified by measuring the size, PDI value and zeta potential (Table 1), and the liposomes were visualized by TEM.

**Fig. 2:**
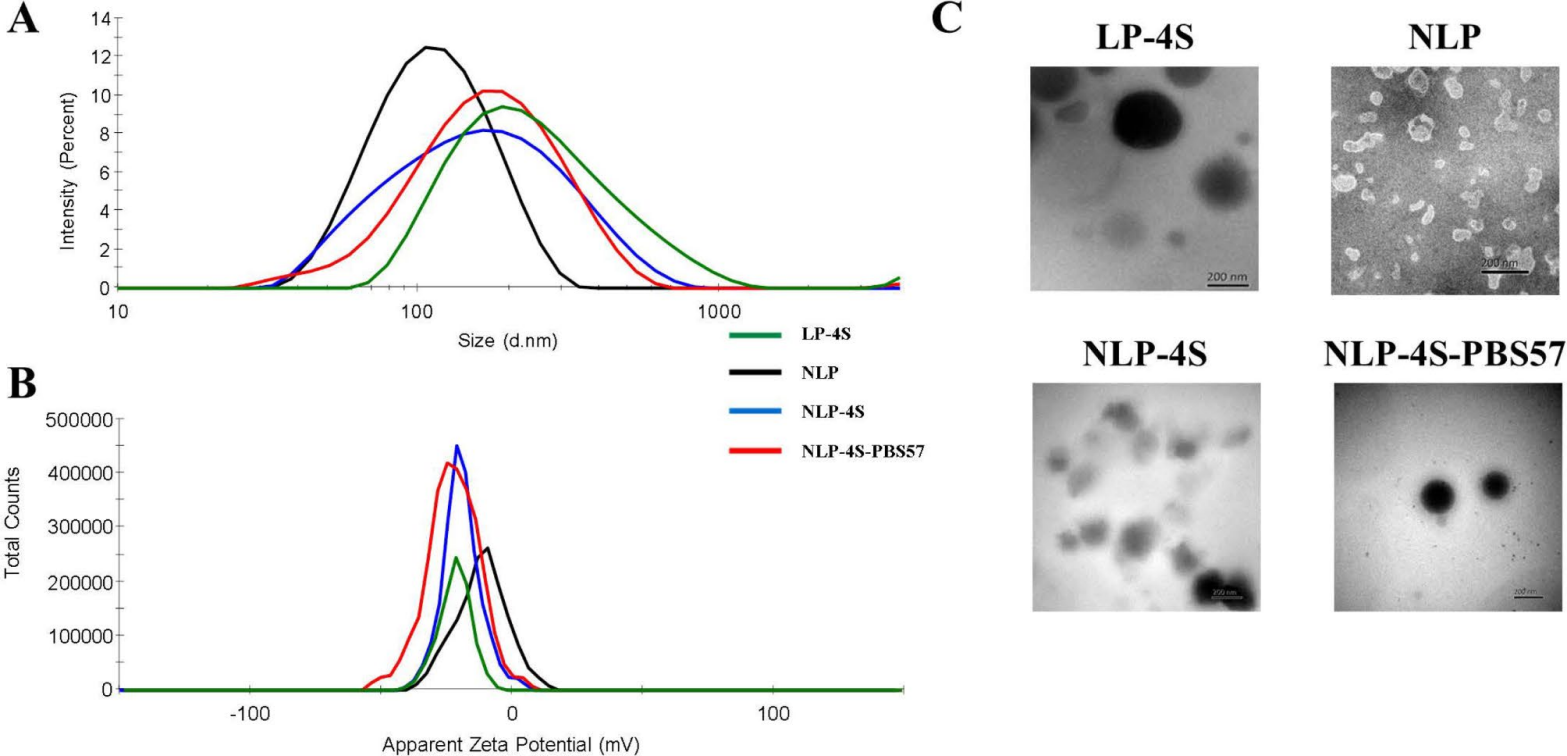
Characterization of liposome vaccines. **A** Particle size distribution of liposomal nanoparticles. DLS measurements of **LP-4S**, **NLP**, **NLP-4S** and **NLP-4S-PBS57** liposomes. **B** Zeta potential of the four liposome vaccines. **C** Representative TEM images of liposome vaccines. Liposomal nanoparticles have basically round-shape structures. **NLP** liposomes were treated by negative staining method, and **LP-4S**, **NLP-4S** and **NLP-4S-PBS57** liposomes were dyed by positive staining method. Scale bar indicates 200 nm

The particle size of liposomes affects their distribution and stability in the blood, and it alters their ability to disclose and present antigens [38]. Thus, liposome-based vaccine delivery systems with suitable dimensions will facilitate the direct interaction of antigens with follicular B cells. In the present study, the hydrodynamic diameters of different types of liposomal particles were measured by DLS (Fig. 2A; Table 1). Each particle size curve line of the four different liposomes, i.e., **LP-4S** (4S-loaded DPPC liposome), **NLP** (blank DOPC liposome), **NLP-4S** (4S-loaded DOPC liposome) and **NLP-4S-PBS57** (4S- and PBS57-loaded DOPC liposome), presented only one peak, indicating that the dispersions of these liposomes were relatively uniform. Specifically, the size of three new formulated liposomes was changed corresponding to the loading of 4S or 4S and PBS57. The original mean particle size of the blank liposome (**NLP**) was 99.3 ± 0.7 nm. The mean particle size of **NLP-4S** reached 126.6 ± 1.1 nm after loading antigen 4S, and the liposomal particle size of **NLP-4S-PBS57** increased to 152.9 ± 6.9 nm after loading both 4S and PBS57. Remarkably, the average diameters of the three newly formulated liposomes were all less than 200 nm, while that of **LP-4S** was 236.8 ± 0.46 nm, suggesting that DOPC liposomes may be a better carrier for antigen 4S.

The PDI value indicates the size distribution of a dispersion system, ranging from 0 to 1.0, in which a high PDI value (> 0.6) suggests a broad size distribution or the presence of large droplets and aggregates [39]. As summarized in Table 1, the PDI values of the newly formulated liposomes **NLP**, **NLP-4S** and **NLP-4S-PBS57** were approximately 0.184, 0.157 and 0.267, respectively, which were significantly lower than that of the preliminary liposome (**LP-4S**) with a PDI value of 0.361. Thus, these findings suggested that all four liposomes have uniform particle sizes and that three **NLP** liposomes may possess excellent stability in blood.

Charge, represented by zeta potential, is important for the stability and encapsulation efficiency of liposomes. As the potential increases, the repulsion between particles becomes larger, resulting in a more stable colloidal dispersion system [40]. The CDG biomarker bears a negatively charged tetrasaccharide (mainly attributed to the terminal sialic acid); thus, the zeta potential values of liposomes would change after loading the antigen. As expected, the least negative zeta potential was − 11.7 ± 1.5 mV for **NLP**, and the most negative zeta potential was − 22.5 ± 1.9 mV for **NLP-4S-PBS57** (Fig. 2B; Table 1). The negative charge on the liposomal surfaces significantly increased after loading 4S, implying that the antigen was successfully inserted into the liposomes. Three liposomal particles carrying antigen 4S afforded similar zeta potential values of -21.8 ± 0.8 mV (**LP-4S**), -19.2 ± 1.8 mV (**NLP-4S**) and − 22.5 ± 1.9 mV (**NLP-4S-PBS57**), suggesting that the zeta potential is not dependent on the lipid composition or addition of the adjuvant (Table 1).

The size and shape of the **LP-4S**, **NLP**, **NLP-4S** and **NLP-4S-PBS57** liposomes were also observed by TEM. As shown in Fig. 2C, all liposomes were nearly spherical. Similar to our previous result of **LP-4S**, a visible color shift to gray‒black was observed after loading 4S, indicating the successful anchoring of antigen in liposomes.

## Mouse immunization and analysis of the immune response against liposomal vaccines

**Fig. 3:**
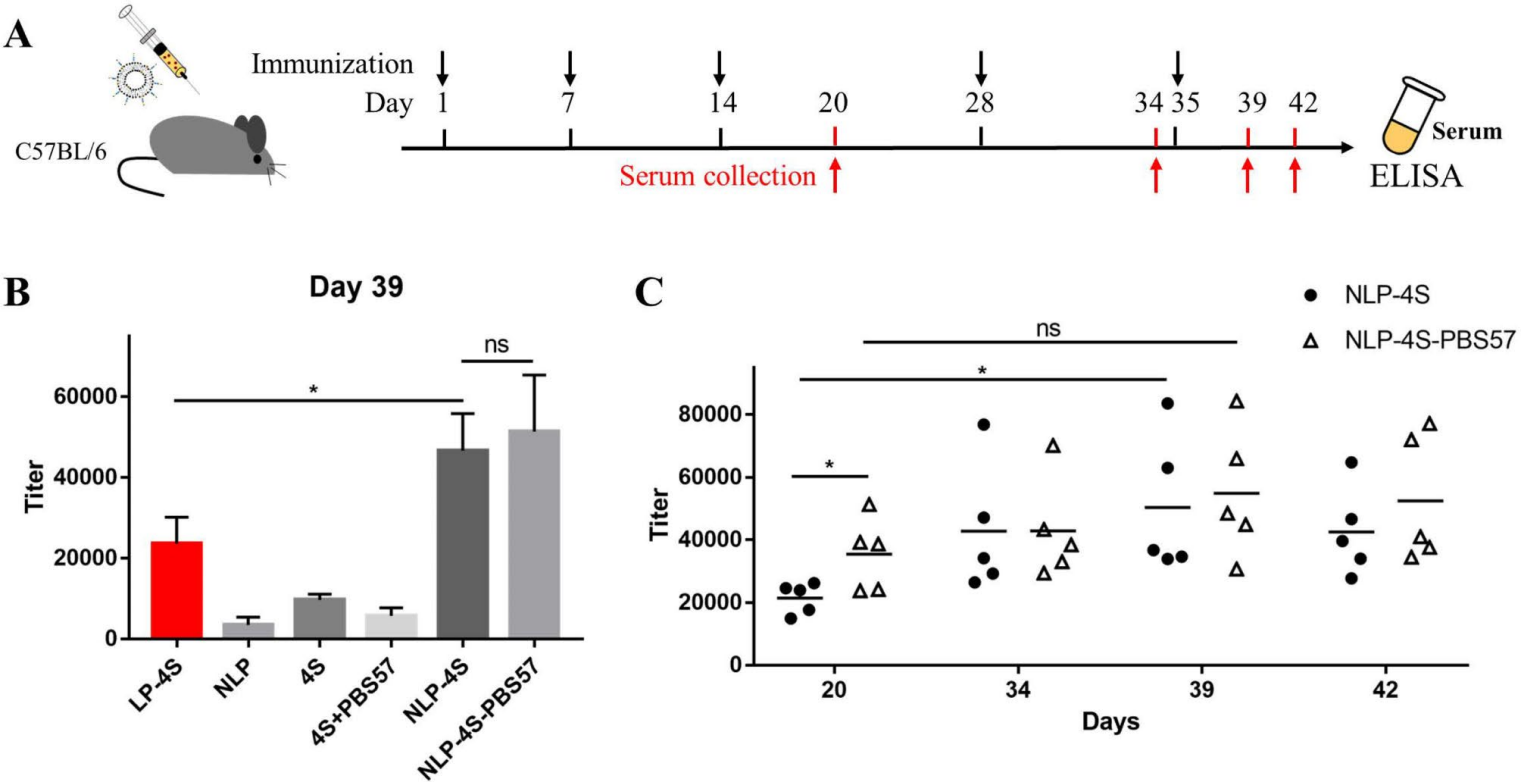
Analysis of the immune response in liposome-vaccinated mice. **A** Schematic representation of the treatment schedule (immunization and sample collection). Female C57BL/6 mice were immunized s.c. at Days 1, 7, 14, 28 and 35. Sera were collected at Days 20, 34, 39 and 42 after vaccination. **B** Antibody titers of Day 39 sera. 4S was used as the coating antigen. Each data reflects an average of three measurements using pooled sera from each of the six groups (Day 39) and goat anti-mouse Ig(G + M). Error bars represent the standard error. **C** Antibody titers elicited by **NLP-4S** and **NLP-4S-PBS57**. 4S was used as the coating antigen. Each line represents the average of sera from Days 20, 34, 39 and 42 from 5 replicate mice (each dot) and goat anti-mouse Ig(G + M). Statistical differences were determined by two-tailed unpaired *t* test analysis and is indicated as either non-significant (ns) or *0.01 < P < 0.05

To determine the effects of the newly formulated liposomal particles and the PBS57 NKT cell agonist, C57BL/6 mice were used for the immunization research. As shown in Fig. 3A, a total of five immunizations on Days 1, 7, 14, 28 and 35 were designed to ensure the effect of immunostimulation. To a group of five female mice, four types of liposomes (**LP-4S**, **NLP**, **NLP-4S** and **NLP-4S-PBS57**) and antigen 4S alone or admixed with PBS57 were each injected subcutaneously. Sera were collected from each mouse by bleeding on Days 20, 34, 39 and 42 (Fig. 3A). The blood samples on Day 0 before the initial immunization were used for baseline measurement, and samples were taken three times (Days 34, 39 and 42) after the fourth and fifth immunizations to confirm the serum antibody concentration reaching the plateau. Under standard protocols, antisera were prepared and stored for the further analysis of CDG biomarker antibodies.

The serum anti-biomarker antibody titers were determined by ELISA. In our preliminary work, the antibody concentration reached a plateau after the fourth injection [21]. Therefore, six groups of mouse antisera after the fifth injection (Day 39) were analyzed (Fig. 3B). The ELISA results of Ig(G + M) antibody titers obtained with 4S alone (4S) or adjuvant with PBS57 (4S + PBS57) revealed that the antigen itself provoked a weak immune response in mice (Fig. 3B), which was similar to the results in a previous study [21]. The addition of PBS57 (4S + PBS57) did not improve the stimulation of the immune response, which may be attributed to the anergy of NKT cells after several times administration of high amount agonist PBS57 [41–44]. Among the four groups injected with liposome vaccines, the blank liposome **NLP** showed a trace effect that was lower than that of the 4S group, suggesting that the generated antibody was specific to the tetrasaccharide antigen rather than the liposome. However, the immunogenicity of 4S was significantly improved when it was assembled into liposomes. As previously reported, liposome **LP-4S** exhibited a moderate immune response, while mice vaccinated with **NLP-4S** produced higher titers (approximately 2 times) of Ig(G + M) antibodies than **LP-4S** regardless of whether PBS57 was introduced into the liposome (Fig. 3B). This result indicated that the 4S antigen remained stable and was more easily caught by immune cells in vivo when loaded into newly formulated DOPC liposomes. The antibody titers stimulated by **NLP-4S** and **NLP-4S-PBS57** were comparable, suggesting that the loading of PBS57 did not significantly affect the stability of liposomes in blood and further immune activation.

## The NKT cell agonist PBS57 induces a rapid immune response and antibody class switching

Our previous study detected that the antibody concentration of **LP-4S** liposomes reached a plateau after the fourth injection [21]. To study the time-immune response correlation of newly formulated liposomes, the total antibody titers of **NLP-4S** and **NLP-4S-PBS57** on Days 20, 34, 39 and 42 were detected by ELISA (Fig. 3C). Similar to the **LP-4S** group, the **NLP-4S** group showed increased antibody titers throughout the immunization boost injections and reached a plateau after the fourth injection (Day 34). The total antibody titers after the fifth injection (Day 39) were significantly higher than those after the third injection (Day 20), which demonstrated that the additional boost injections were necessary. In contrast, the **NLP-4S-PBS57** group reached the plateau faster, and the total antibody level on Day 20 was significantly higher than that in the **NLP-4S** group but remained almost the same after the fourth and fifth injections (Fig. 3C). These findings demonstrated that the adjuvanticity of the NKT cell agonist PBS57 induces a faster immune response. For the **NLP-4S-PBS57** liposomes, three or four injections are sufficient to elicit a robust immune response.

**Fig. 4:**
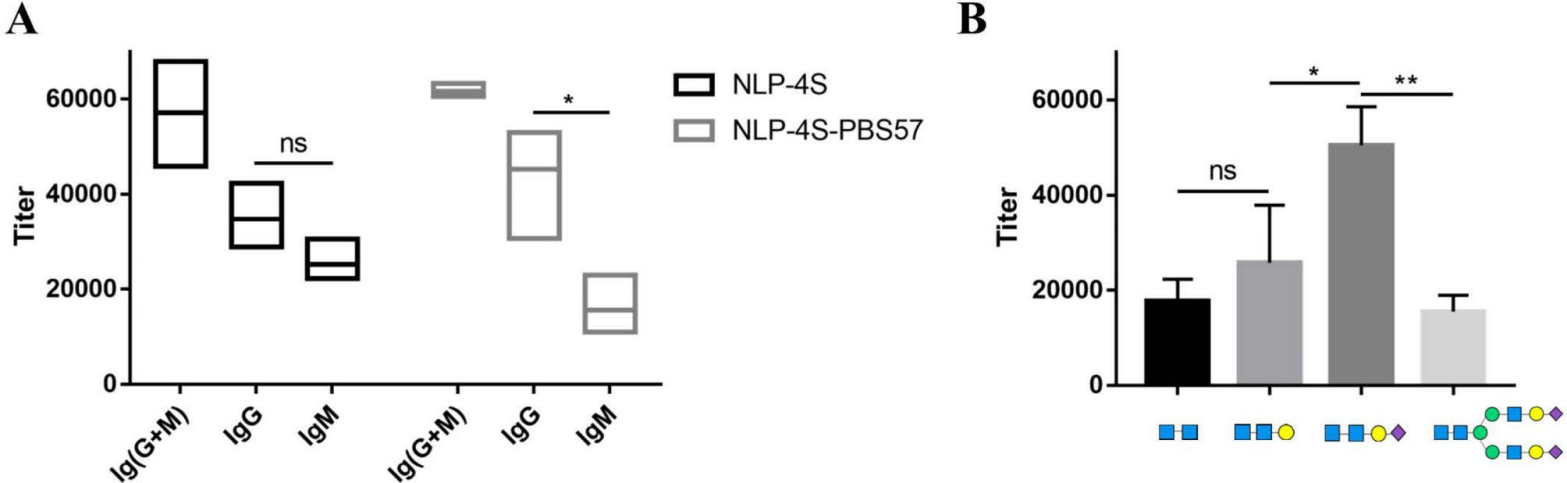
Analysis of the subtype and specificity of antibody. **A** Analysis of the antibody subtype (IgG and IgM). 4S was used as the coating antigen. Each data reflects the ranges of three measurements using pooled sera from the **NLP-4S** and **NLP-4S-PBS57** groups (Day 39) and goat anti-mouse Ig(G + M), IgG or IgM. The lines represent the mean values. **B** Analysis of antibody specificity. Disaccharide (GlcNAc2-PP-Phy), trisaccharide (GalGlcNAc2-PP-Phy), 4S (SiaGalGlcNAc2-PP-Phy) and biantennary complex type N-glycan (SiaGalGlcNAc)2Man3GlcNAc2-AsnFmoc were used as the coating antigens respectively. Each data reflects an average of three measurements using pooled sera from the **NLP-4S-PBS57** (Day 39) and goat anti-mouse IgG. Error bars represent the standard error. Statistical differences were determined by two-tailed unpaired *t* test analysis and is indicated as non-significant (ns), *0.01 < P < 0.05 or **0.001 < P ≤ 0.01

As expected, **LP-4S** induced almost equal levels of IgM and IgG, indicating that it elicited only an ordinary long-term immune response and modest affinity antibodies [45]. To develop a diagnostic method for ALG1- and PMM2-CDG, the IgG specific antibody, which has strong affinity and a long half-life, is desired. Therefore, we attempted to enhance IgG production through antibody class switching by adding PBS57 as the adjuvant, and analyzed the types of antibodies from mice vaccinated with **NLP-4S** and **NLP-4S-PBS57** on Day 39 (Fig. 4A). For the **NLP-4S** group, IgG and IgM antibodies were produced at almost equal level, which was similar to **LP-4S**, which demonstrated that the alteration of phosphatidylcholine in liposomes did not promote antibody class switching. In contrast, mice vaccinated with **NLP-4S-PBS57** showed a significantly higher titer of IgG than IgM (Fig. 4A), suggesting that the newly formulated liposome stimulated the immune response with long-term memory. These results further confirmed that PBS57 induces the antibody switch from IgM to IgG, which is consistent with the known function of PBS57 in immunology [46, 47].

## NLP-4S-PBS57 stimulates CDG biomarker-specific IgG

The antigenic specificity of the antibodies determines whether they are applicable for further study and application. Consequently, the specificity of the IgG antibody stimulated by **NLP-4S-PBS57** was detected with ELISA (Fig. 4B). The binding affinity of antisera against GalGlcNAc2, GlcNAc2 (truncated glycan structures lacking terminal Sia or Sia-Gal capping) and (SiaGalGlcNAc)2Man3GlcNAc2 (a typical biantennary complex type N-glycan) were compared with the SiaGalGlcNAc2 tetrasaccharide antigen. IgG recognized and bound to the SiaGalGlcNAc2 CDG biomarker with higher titers (2–3 times) than other glycan structures. These results suggested that **NLP-4S-PBS57** can be used to stimulate the production of antigen-specific IgG antibodies.

## Characterization of the IgG subclass response to the newly formulated liposomes

**Fig. 5:**
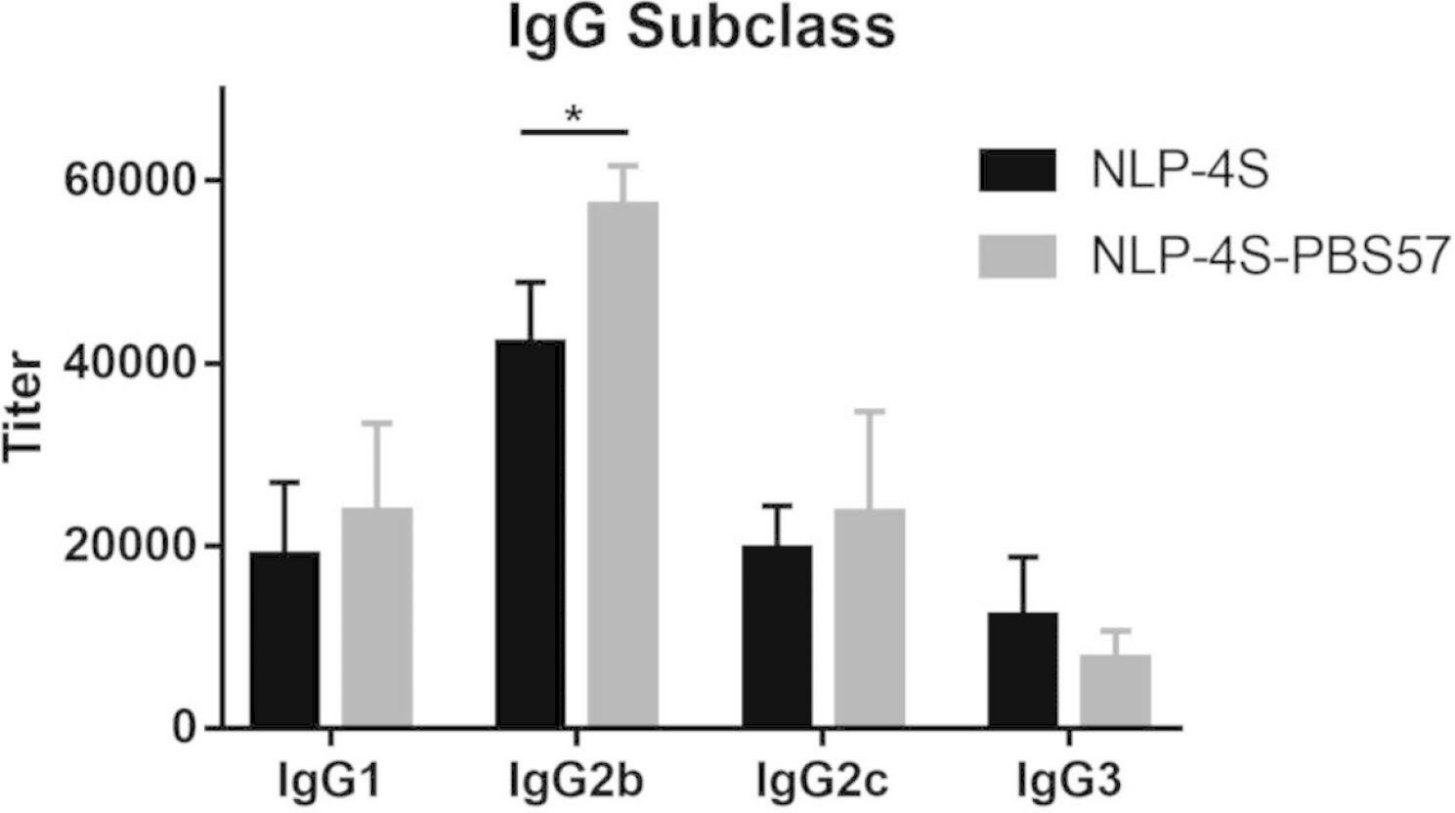
Analysis of the IgG antibody subclasses elicited by **NLP-4S** and **NLP-4S-PBS57**. 4S was used as the coating antigen. Each data reflects an average of three measurements using pooled sera from the **NLP-4S** and **NLP-4S-PBS57** groups (Day 39) and goat anti-mouse IgG1, IgG2b, IgG2c and IgG3 antibodies. Error bars represent the standard error. Statistical differences were determined by two-tailed unpaired *t* test analysis and is indicated as non-significant (ns) or *0.01 < P < 0.05

In addition to total antibodies and antibody isotypes, IgG subclasses, such as IgG1, IgG2b, IgG2c and IgG3, stimulated by **NLP-4S** and **NLP-4S-PBS57** were also individually assessed by ELISA. Both groups of mice exhibited similar immunologic responses, namely, they produced high levels of IgG2b antibody and moderate IgG1, IgG2c and IgG3 antibodies (Fig. 5). The results were consistent with previous reports that glycolipids carrying liposomal vaccines may elicit antigen-specific IgG2 antibodies [25]. Therefore, these results demonstrated that the newly formulated liposomes, **NLP-4S** and **NLP-4S-PBS57**, elicit robust NKT cell-dependent B cell responses in mice. Moreover, compared to **NLP-4S**, **NLP-4S-PBS57** liposomes elicited a slightly but statistically distinguishable higher IgG2b antibody titer, while the IgG1, IgG2c and IgG3 subclasses of IgG antibodies showed no or little response to the PBS57 adjuvant.

## Discussion

Based on our preliminary work, the major challenge for CDG biomarker vaccines is to induce high titers of specific IgG antibodies [21]. To address this issue, we redesigned liposomal formulation and added the PBS57 adjuvant. First, the phosphatidylcholine component was changed from DPPC to DOPC. The original DPPC bears fully saturated acyl chains (Fig. 1A), which cause the DPPC bilayer to be in the gel phase and barely diffuse at room temperature. Thus, DPPC liposomes have to be prepared at a higher temperature (41 ℃, main phase transition temperature), which may harm glycolipid antigens [34]. DOPC shares similar biochemical properties with DPPC but possesses unsaturated acyl chains (Fig. 1A), which allows DOPC to exist in a fluid phase at room temperature. The DOPC liposomes could be prepared easily due to the diffusibility of DOPC molecules, and this feature is of great significance to maintain the stability of the 4S antigen and PBS57 adjuvant.

Currently, several liposomal formulations have been approved for human use or have reached advanced clinical development to produce vaccines [48, 49]. It is known that nanoparticles with diameters less than 200 nm can reach the lymphoid organs directly through lymph drainage [50], and those with virus-like dimensions (approximately 100 nm) most efficiently reach the lymph nodes in a cell-free state [51, 52]. In addition, smaller liposomes (diameters < 100 nm) are more likely to be degraded and ultimately lead to a weaker immune response, whereas the transport of larger liposomal particles (diameters 200–500 nm) requires dendritic cells and takes a long time (approximately 24 h) to arrive in lymph nodes [38, 53]. Thus, we aimed to prepare liposomes manufactured at suitable diameters (100–200 nm), which may improve lymph node targeting. By optimizing the phosphatidylcholine structure and proportion (Table 1), the diameters of the newly formulated **NLP** blank liposomes were determined to be approximately 100 nm (99.3 ± 0.7 nm). After loading the 4S antigen with or without the PBS57 adjuvant, the liposomal particle sizes increased to 126.6 ± 1.1 nm (**NLP-4S**) and 152.9 ± 6.9 nm (**NLP-4S-PBS57**), respectively (Fig. 2). The sizes of the two DOPC liposomes were 100–200 nm, indicating that they were capable of transporting antigens more efficiently than the original DPPC liposome **LP-4S** (diameter 236 nm). In addition to particle sizes, other parameters of liposomes, such as PDI, were measured, which indicated that the DOPC liposomes possessed excellent stability in blood. As expected, the ELISA results showed that **NLP-4S** and **NLP-4S-PBS57** stimulated higher antibody titers than **LP-4S** (Fig. 3B), which may have due to their in vivo stability and motility.

**LP-4S** liposomes have been previously reported to induce only a modest long-term immune response, generating high titers of IgM rather than IgG after four immunizations [21]. To address the challenge of generating a high level of IgG, we employed the NKT cell agonist, PBS57, as an adjuvant. As a galactosylceramide analog, PBS57 stimulates glycolipid presentation by activating NKT cells [32]. It has recently been reported that PBS57-stimulated NKT cells provide T cell help to B cells and exhibit antibody class switching from IgM to IgG [46, 47]. Moreover, PBS57 inserts into the membrane of liposomes with lipid chains, similar to the tetrasaccharide antigen 4S, resulting in the advantage of **NLP-4S-PBS57** liposomes presenting oligosaccharide epitopes together with NKT cell agonists. In the present study, substantially increased IgG titers were observed after the fourth vaccination with **NLP-4S-PBS57** (Fig. 4A), demonstrating the enhancement of B cell immune responses. As shown in Figs. 3B and 4B, the generated antibodies specifically responded to the targeted carbohydrate epitopes rather than liposomes or other glycan structures. Typically, the terminal sialylated biantennary complex type N-glycan, which bears Sia-Gal and is widely distributed in mammalian glycoproteins, was hardly recognized by IgG from mice sera (Fig. 4B). The antibody concentration and specificity against tetrasaccharide biomarker were also confirmed by ELISA using tetrasaccharide conjugated bovine serum albumin as coating antigen [54], which showed similar results as coating the plates with 4S (data not shown).

The IgG antibody subtype levels on Day 39 were further determined to reveal the effect of adjuvant PBS57. The subtypes of IgG are highly conserved in structure and defined according to their constant regions, while they play different roles to activate the immune system [55]. In the sera of C57BL/6 mice, there are four IgG subtypes, IgG1, IgG2b, IgG2c and IgG3 [56, 57]. **NLP-4S** and **NLP-4S-PBS57** liposomes elicited similar patterns of immune response, namely, they both induced mainly IgG2b, IgG2c, IgG1, and some IgG3 antibodies (Fig. 5). Unlike other subtypes, the level of IgG2b antibody increased markedly after the addition of PBS57 compared with **NLP-4S**. Among the IgG subtypes, the activity hierarchy is in the order of IgG2 > IgG1 ≫ IgG3, and IgG2b is the most potent for effector response activation [58]. Glyco-antigens usually induce high levels of IgG2 antibodies and require the addition of adjuvants [59], which is consist with our results that **NLP-4S-PBS57** induced higher level of IgG2b than **NLP-4S**. These results exhibited that the newly formulated liposomes with adjuvant PBS57 led to strong T cell-dependent immunity, suggesting their potential as vaccines against ALG1- and PMM2-CDG biomarker.

## Conclusion

In the present study, we described a reformulated liposomal nanoparticle platform based on an adjuvant with the NKT cell agonist, PBS57. Using this platform, carbohydrate vaccines carrying ALG1- and PMM2-CDG biomarker tetrasaccharides were prepared. The newly formulated DOPC liposomes could be manufactured at more suitable diameters and showed excellent uniformity and stability. Immunization with these DOPC liposomes stimulated a strong immune response and generated high titers of antibodies. In particular, **NLP-4S-PBS57** induced rapid immunostimulation and induced the antibody class switching to oligosaccharide epitope-specific IgG. Comparison of the IgG production in response to vaccination with **NLP-4S** and **NLP-4S-PBS57** suggested that the NKT cell antigen PBS57, which elicits strong T cell-dependent immunity, is important in the liposomal formulation. These results provided evidence that the reformulated liposomal platform is suitable for the production of anti-CDG biomarker antibodies, which may have significance for developing diagnostic methods for ALG1- and PMM2-CDG.

## Methods

### Materials and general methods

Chemicals and materials were obtained from our laboratory or commercial sources and were used as received without further purification unless otherwise noted. All the solutions were prepared fresh. DOPC was purchased from Avanti Polar Lipids (AL, USA), and CH was purchased from Sangon Biotech (Shanghai, China). The PBS57 glycosylceramide was purchased from Chemsky International Co., Ltd. (Shanghai, China). UDP-galactose (UDP-Gal) was purchased from Santa Cruz Biotechnology (CA, USA), and CMP-N-acetylneuraminic acid (CMP-NANA) was purchased from Carbosynth Ltd. (Berkshire, UK). Citronellol was purchased from Adamas Reagent (Basel, Switzerland). Other reagents were purchased from Sangon Biotech (Shanghai, China), Sigma‒Aldrich (MO, USA) or Tokyo Chemical Industries Co., Ltd. (Tokyo, Japan). Goat anti-mouse IgG H&L (Alkaline Phosphatase) (Catalog No. ab97020), goat anti-mouse IgM mu chain (Alkaline Phosphatase) preadsorbed (Catalog No. ab98672), goat anti-mouse IgG1 (horse radish peroxidase, HRP) preadsorbed (Catalog No. ab98693), goat anti-mouse IgG2b heavy chain (HRP) (Catalog No. ab97250), goat anti-mouse IgG2c heavy chain (HRP) (Catalog No. ab97255) and goat anti-mouse IgG3 heavy chain (HRP) (Catalog No. ab97260) were obtained from Abcam (Cambridge, UK). Goat anti-mouse IgG (H + L) and HRP conjugate (Catalog No. HS201) were purchased from TransGen Biotech (Beijing, China). The alkaline phosphatase yellow (pNPP) liquid substrate system for ELISA (Catalog No. P7998) was purchased from Sigma‒Aldrich (MO, USA). Tetramethyl benzidine (TMB) solution for enzyme-linked immunosorbent assay (ELISA) (Catalog No. P0209) was purchased from Beyotime Biotech (Shanghai, China). Dulbecco’s Phosphate-Buffered (Catalog No. E607009) was purchased from Sangon Biotech (Shanghai, China).

Matrix-assisted laser desorption ionization (MALDI) time-of-flight (TOF) mass spectrometry was measured by using an UltrafleXtreme from Bruker Scientific LLC (MA, USA). Thin layer chromatography (TLC) analysis was performed on Merck 60 F_254_ silica-coated plates from Millipore (MA, USA). Dynamic light scattering (DLS) analysis was performed on a Zetasizer Nano ZS (ZEN3700) from Malvern Instruments Ltd. (Malvern, UK). The transmission electron microscope (TEM) H-7650 was from Hitachi (Tokyo, Japan). The ELISA data were obtained by coating the antigens on Nunc MaxiSorp flat-bottom plates from Thermo Fisher Scientific (MA, USA) and measured with an iMark Microplate Reader from Bio-Rad (CA, USA).

### Synthesis of LLO 4S

LLO 4S was synthesized using a strategy similar to previous reports [21, 60]. Chemically synthesized phytanyl pyrophosphoryl chitobioside (GlcNAc2-PP-Phy) was used as the substrate for the successive addition of galactose and sialic acid residues using optimized reaction conditions. Recombinant human β-1,4-galactosyltransferase (*h*GalT) and *Photobacterium damsla* α-2,6-sialyltransferase (*Pd*SiaT) were expressed in *Escherichia coli* and purified. Galactosylation and sialylation reactions were performed in MES/NaOH buffer (50 mM, pH 7.4), 10 mM MgCl_2_ and 0.1% NP-40. In brief, GlcNAc2-PP-Phy was incubated with UDP-Gal (6 eq.) in the presence of *h*GalT at 37 °C for 12 h to complete the galactosylation reaction followed by the in situ addition of *Pd*SiaT and CMP-NANA (7 eq.), and the reaction lasted for another 0.5 h at 37 °C. The reaction was analyzed by MALDI-TOF and TLC, which showed the complete formation of sialylated LLO 4S. The reaction was then quenched by adding ethanol at 4 °C and stirred for 0.5 h, and proteins were removed by centrifugation (8,000 × *g*, 20 min). The supernatant was freeze dried, and the residues were extracted by methanol and chloroform (1:2, v/v). The organic phase containing the 4S product was dried with a nitrogen stream and directly applied to the liposome preparation.

### Preparation of liposomes

Liposomes were prepared by a thin film hydration ultrasonic method following reported procedures [35, 37]. **LP-4S** was prepared as previously described [21]. For reformulated liposomes, **NLP-4S** was prepared as follows: DOPC, CH and 4S were dissolved in chloroform and methanol (2:1, v/v) at a molar ratio of 10:5:1 and stirred until uniformly mixed at room temperature; the mixture was evaporated to remove the organic solvent, forming a uniform film on the bottom of the flask; the film was completely hydrated with HEPES buffer (20 mM, pH 7.4) and sonicated for 15 min (5 s on and 5 s off) at 4 °C to give liposomal nanoparticles (**NLP-4S**). The preparation of **NLP** was achieved using the above conditions with a molar ratio of DOPC, CH and phytanyl phosphate (Phy-P) of 4:2:1. The same conditions were applied to prepare **NLP-4S-PBS57** liposomes, which were constructed by DOPC, CH, 4S and PBS57 in a molar ratio of 40:20:4:1. The liposomal nanoparticles were stored at 4 ℃ before use.

### Characterization of liposomes

Particle size and zeta potential were measured using DLS with a Zetasizer Nano ZS by distributing samples three times at 25 ℃ and 90° detector angles. A dispersion of the liposomes (5 mg/mL, 10 µL) was diluted with 3 mL of HEPES (20 mM, pH 7.4) to measure the liposomal particle size. To measure the zeta potential, liposomes (0.1 mg/mL) were dispersed in HEPES (20 mM, pH 7.4) and loaded in a capillary cell mounted on the apparatus. Measurements were performed three times to obtain the mean value.

To observe the morphology of the liposome, a drop of the liposome dispersion (0.1 mg/mL) was placed on a 100-mesh copper grid, and the excess dispersion was then removed with a piece of filter paper. A 2% phosphotungstic acid solution (pH 7.4) was dropped on the grid and dried in a desiccator for 12 h. The morphology of the resulting cationic liposomes was examined with TEM H-7650.

### Animal immunization

Specific pathogen-free C57BL/6 mice (5–6 weeks old, female, Shanghai Slac Laboratory Animal Co., Ltd., China) were randomly divided into groups (5 mice/experimental group) and acclimatized for one week before starting the study. Each group of mice was inoculated with subcutaneous (s.c.) injection (100 µL per mouse) of various liposomes, including blank liposomes (**NLP**), liposomes bearing 12 µg of antigen 4S (**LP-4S** and **NLP-4S**) and liposomes bearing 12 µg of antigen 4S and 4 µg of adjuvant PBS57 (**NLP-4S-PBS57**). In addition, another two groups of mice were injected with 12 µg of 4S and 4 µg of PBS57 mixed with 12 µg of 4S for comparison. Normal mouse sera were collected one day prior to the first injection (Day 0) as the baseline. Booster injections were administered on Days 7, 14, 28 and 35 following the immunization schedule. Mice were bled on Days 20, 34, 39 and 42 to collect 100 µL of whole blood through the tail vein from each mouse, which was stored at 4 °C for 12 h. After centrifugation (5,000 × *g*, 5 min, 4 °C), sera were collected and stored at -80 °C before use.

### Analysis of the antibody response by ELISA

ELISA plates (96-well) were coated with synthetized LLOs, including 4S (2 µg/mL, 100 µL/well), dissolved in bicarbonate solution (0.1 M, pH 9.6) and kept at 37 °C for 1 h. After washing three times with phosphate-buffered saline (PBS) containing 0.05% Tween-20 (PBST), the plates were blocked with blocking buffer (1% citronellol in PBS, 200 µL/well) and incubated at room temperature for 1 h. The plates were then washed three times with PBST and then incubated with an individual mouse serum or pooled sera (100 µL/well) with a dilution ranging from 1:900 to 1:72900 in PBS at 37 °C for 2 h. The plates were then washed three times with PBST.

When measuring the mouse antiserum titers with AP-linked secondary antibodies, the plates were incubated with AP-linked goat anti-mouse Ig (G + M), IgG or IgM antibody (1:1000 dilution in PBS, 100 µL/well) at room temperature for 1 h. The plates were washed three times with PBST and incubated with pNPP solution (100 µL/well) at room temperature for 0.5 h. The optical density (OD) at 405 nm was measured using an iMark Microplate Reader. When measuring the mouse antiserum titers with HRP-linked secondary antibodies, the plates were incubated with HRP-linked goat anti-mouse IgG1, IgG2b, IgG2c and IgG3 antibodies (1:5000 dilution in PBS, 100 µL/well) at room temperature for 2 h. The plates were washed three times with PBST and incubated with TMB solution (100 µL/well) at room temperature for 15 min in the dark. The reaction was stopped by the addition of a H_2_SO_4_ solution (2 M, 100 µL/well), and the OD at 450 nm was measured. The OD values after deducting the background OD of the Day 0 sera were plotted against the logarithmic scale of antiserum dilution values, and a best-fit line was obtained. The equation of the line was employed to calculate the dilution value at which an OD of 0.05 was achieved (endpoint titer), and the antibody titer was obtained as the inverse of the dilution value.

### Statistical analysis

Pooled serum ELISA experiments were independently repeated three times. The sample size, mean and standard deviation (SD) value are provided in the figures. All statistical analyses were performed using GraphPad Prism software (version 8.3.0, CA, USA). Statistical differences between groups were determined using two-tailed unpaired *t* test analysis, and p < 0.05 was considered a statistically significant difference.

## Data Availability

The datasets used and/or analysed during the current study are available from the corresponding author on reasonable request.

## References

[1] Verheijen J, Tahata S, Kozicz T, Witters P, Morava E (2020). Therapeutic approaches in congenital Disorders of Glycosylation (CDG) involving N-linked glycosylation: an update. Genet Med.

[2] Francisco R, Marques-da-Silva D, Brasil S, Pascoal C, Dos Reis Ferreira V, Morava E, Jaeken J (2019). The challenge of CDG diagnosis. Mol Genet Metab.

[3] Cherepanova N, Shrimal S, Gilmore R (2016). N-linked glycosylation and homeostasis of the endoplasmic reticulum. Curr Opin Cell Biol.

[4] Roth J, Zuber C, Park S, Jang I, Lee Y, Kysela KG, Le Fourn V, Santimaria R, Guhl B, Cho JW (2010). Protein N-glycosylation, protein folding, and protein quality control. Mol Cells.

[5] Vilas A, Yuste-Checa P, Gallego D, Desviat LR, Ugarte M, Perez-Cerda C, Gamez A, Perez B (2020). Proteostasis regulators as potential rescuers of PMM2 activity. Biochim Biophys Acta Mol Basis Dis.

[6] Gao XD, Nishikawa A, Dean N (2004). Physical interactions between the Alg1, Alg2, and Alg11 mannosyltransferases of the endoplasmic reticulum. Glycobiology.

[7] Albright CF, Robbins RW (1990). The sequence and transcript heterogeneity of the yeast gene ALG1, an essential mannosyltransferase involved in N-glycosylation. J Biol Chem.

[8] Matthijs G, Schollen E, Pardon E, Veiga-Da-Cunha M, Jaeken J, Cassiman JJ, Van Schaftingen E (1997). Mutations in PMM2, a phosphomannomutase gene on chromosome 16p13, in carbohydrate-deficient glycoprotein type I syndrome (Jaeken syndrome). Nat Genet.

[9] Zhang W, James PM, Ng BG, Li X, Xia B, Rong J, Asif G, Raymond K, Jones MA, Hegde M (2016). A novel N-Tetrasaccharide in patients with congenital Disorders of Glycosylation, including asparagine-linked glycosylation protein 1, phosphomannomutase 2, and mannose phosphate isomerase deficiencies. Clin Chem.

[10] Quelhas D, Quental R, Vilarinho L, Amorim A, Azevedo L (2007). Congenital disorder of glycosylation type Ia: searching for the origin of common mutations in PMM2. Ann Hum Genet.

[11] Moravej H, Altassan R, Jaeken J, Enns GM, Ellaway C, Balasubramaniam S, De Lonlay P, Coman D, Mercimek-Andrews S, Witters P, Morava E (2020). Hypoglycemia in CDG patients due to PMM2 mutations: follow up on hyperinsulinemic patients. JIMD Rep.

[12] Ng BG, Shiryaev SA, Rymen D, Eklund EA, Raymond K, Kircher M, Abdenur JE, Alehan F, Midro AT, Bamshad MJ (2016). ALG1-CDG: clinical and molecular characterization of 39 unreported patients. Hum Mutat.

[13] Peanne R, de Lonlay P, Foulquier F, Kornak U, Lefeber DJ, Morava E, Perez B, Seta N, Thiel C, Van Schaftingen E (2018). Congenital disorders of glycosylation (CDG): Quo vadis?. Eur J Med Genet.

[14] Al Teneiji A, Bruun TU, Sidky S, Cordeiro D, Cohn RD, Mendoza-Londono R, Moharir M, Raiman J, Siriwardena K, Kyriakopoulou L, Mercimek-Mahmutoglu S (2017). Phenotypic and genotypic spectrum of congenital disorders of glycosylation type I and type II. Mol Genet Metab.

[15] Mousa J, Veres L, Mohamed A, De Graef D, Morava E (2022). Acetazolamide treatment in late onset CDG type 1 due to biallelic pathogenic DHDDS variants. Mol Genet Metab Rep.

[16] Bruneel A, Cholet S, Drouin-Garraud V, Jacquemont ML, Cano A, Megarbane A, Ruel C, Cheillan D, Dupre T, Vuillaumier-Barrot S (2018). Complementarity of electrophoretic, mass spectrometric, and gene sequencing techniques for the diagnosis and characterization of congenital disorders of glycosylation. Electrophoresis.

[17] Hipgrave Ederveen AL, de Haan N, Baerenfaenger M, Lefeber DJ, Wuhrer M (2020). Dissecting total plasma and protein-specific glycosylation profiles in congenital Disorders of Glycosylation. Int J Mol Sci.

[18] Abu Bakar N, Lefeber DJ, van Scherpenzeel M (2018). Clinical glycomics for the diagnosis of congenital disorders of glycosylation. J Inherit Metab Dis.

[19] Witters P, Edmondson AC, Lam C, Johnsen C, Patterson MC, Raymond KM, He M, Freeze HH, Morava E (2021). Spontaneous improvement of carbohydrate-deficient transferrin in PMM2-CDG without mannose observed in CDG natural history study. Orphanet J Rare Dis.

[20] Bengtson P, Ng BG, Jaeken J, Matthijs G, Freeze HH, Eklund EA (2016). Serum transferrin carrying the xeno-tetrasaccharide NeuAc-Gal-GlcNAc2 is a biomarker of ALG1-CDG. J Inherit Metab Dis.

[21] Jia JX, Kalisa NY, Lu TT, Zhou Z, Gao XD, Wang N (2020). Chemo-enzymatic synthesis of the ALG1-CDG biomarker and evaluation of its immunogenicity. Bioorg Med Chem Lett.

[22] de Morais FAP, Goncalves RS, Vilsinski BH, de Oliveira EL, Rocha NL, Hioka N, Caetano W (2019). Hypericin photodynamic activity in DPPC liposome. PART I: biomimetism of loading, location, interactions and thermodynamic properties. J Photochem Photobiol B.

[23] Khatun F, Toth I, Stephenson RJ. Immunology of carbohydrate-based vaccines. Adv Drug Deliv Rev. 2020;165–166:117 – 26. 10.1016/j.addr.2020.04.006 32320714

[24] Yang F, Zheng XJ, Huo CX, Wang Y, Zhang Y, Ye XS (2011). Enhancement of the immunogenicity of synthetic carbohydrate vaccines by chemical modifications of STn antigen. ACS Chem Biol.

[25] Yao L, Wu L, Wang R, Liu Y, Luo F, Zhang Y, Chen G (2022). Liposome-Based Carbohydrate Vaccine for simultaneously eliciting Humoral and Cellular Antitumor Immunity. ACS Macro Lett.

[26] Rao M, Peachman KK, Alving CR (2021). Liposome Formulations as Adjuvants for vaccines. Curr Top Microbiol Immunol.

[27] Pifferi C, Berthet N, Renaudet O (2017). Cyclopeptide scaffolds in carbohydrate-based synthetic vaccines. Biomater Sci.

[28] Peri F (2013). Clustered carbohydrates in synthetic vaccines. Chem Soc Rev.

[29] Bai L, Deng S, Reboulet R, Mathew R, Teyton L, Savage PB, Bendelac A (2013). Natural killer T (NKT)-B-cell interactions promote prolonged antibody responses and long-term memory to pneumococcal capsular polysaccharides. Proc Natl Acad Sci U S A.

[30] Saez de Guinoa J, Jimeno R, Gaya M, Kipling D, Garzon MJ, Dunn-Walters D, Ubeda C, Barral P (2018). CD1d-mediated lipid presentation by CD11c(+) cells regulates intestinal homeostasis. EMBO J.

[31] Venkataswamy MM, Porcelli SA (2010). Lipid and glycolipid antigens of CD1d-restricted natural killer T cells. Semin Immunol.

[32] Griewank KG, Lorenz B, Fischer MR, Boon L, Lopez Kostka S, von Stebut E (2014). Immune modulating effects of NKT cells in a physiologically low dose Leishmania major infection model after alphaGalCer analog PBS57 stimulation. PLoS Negl Trop Dis.

[33] Maia ML, Pereira CS, Melo G, Pinheiro I, Exley MA, Porto G, Macedo MF (2015). Invariant natural killer T cells are reduced in Hereditary Hemochromatosis Patients. J Clin Immunol.

[34] Szoka F, Papahadjopoulos D (1980). Comparative properties and methods of preparation of lipid vesicles (liposomes). Annu Rev Biophys Bioeng.

[35] Deng S, Bai L, Reboulet R, Matthew R, Engler DA, Teyton L, Bendelac A, Savage PB (2014). A peptide-free, liposome-based oligosaccharide vaccine, adjuvanted with a natural killer T cell antigen, generates robust antibody responses in vivo. Chem Sci.

[36] Yeh HW, Lin TS, Wang HW, Cheng HW, Liu DZ, Liang PH (2015). S-Linked sialyloligosaccharides bearing liposomes and micelles as influenza virus inhibitors. Org Biomol Chem.

[37] Liao G, Zhou Z, Suryawanshi S, Mondal MA, Guo Z (2016). Fully synthetic self-adjuvanting alpha-2,9-Oligosialic acid based Conjugate vaccines against Group C Meningitis. ACS Cent Sci.

[38] Bachmann MF, Jennings GT (2010). Vaccine delivery: a matter of size, geometry, kinetics and molecular patterns. Nat Rev Immunol.

[39] Danaei M, Dehghankhold M, Ataei S, Hasanzadeh Davarani F, Javanmard R, Dokhani A, Khorasani S, Mozafari MR (2018). Impact of particle size and Polydispersity Index on the clinical applications of Lipidic Nanocarrier Systems. Pharmaceutics.

[40] Gopi S, Balakrishnan P (2021). Evaluation and clinical comparison studies on liposomal and non-liposomal ascorbic acid (vitamin C) and their enhanced bioavailability. J Liposome Res.

[41] Singh AK, Gaur P, Das SN (2014). Natural killer T cell anergy, co-stimulatory molecules and immunotherapeutic interventions. Hum Immunol.

[42] Parekh VV, Wilson MT, Olivares-Villagomez D, Singh AK, Wu L, Wang CR, Joyce S, Van Kaer L (2005). Glycolipid antigen induces long-term natural killer T cell anergy in mice. J Clin Invest.

[43] Yin XG, Lu J, Wang J, Zhang RY, Wang XF, Liao CM, Liu XP, Liu Z, Guo J (2021). Synthesis and evaluation of liposomal Anti-GM3 Cancer vaccine candidates covalently and noncovalently adjuvanted by alphaGalCer. J Med Chem.

[44] Iyoda T, Ushida M, Kimura Y, Minamino K, Hayuka A, Yokohata S, Ehara H, Inaba K (2010). Invariant NKT cell anergy is induced by a strong TCR-mediated signal plus co-stimulation. Int Immunol.

[45] Lam JH, Smith FL, Baumgarth N (2020). B cell activation and response regulation during viral infections. Viral Immunol.

[46] Schmid H, Schneidawind C, Jahnke S, Kettemann F, Secker KA, Duerr-Stoerzer S, Keppeler H, Kanz L, Savage PB, Schneidawind D (2018). Culture-expanded human invariant natural killer T cells suppress T-Cell alloreactivity and eradicate leukemia. Front Immunol.

[47] Leadbetter EA, Brigl M, Illarionov P, Cohen N, Luteran MC, Pillai S, Besra GS, Brenner MB (2008). NK T cells provide lipid antigen-specific cognate help for B cells. Proc Natl Acad Sci U S A.

[48] Mai Y, Guo J, Zhao Y, Ma S, Hou Y, Yang J (2020). Intranasal delivery of cationic liposome-protamine complex mRNA vaccine elicits effective anti-tumor immunity. Cell Immunol.

[49] Wang N, Chen M, Wang T (2019). Liposomes used as a vaccine adjuvant-delivery system: from basics to clinical immunization. J Control Release.

[50] Swartz MA (2001). The physiology of the lymphatic system. Adv Drug Deliv Rev.

[51] Cubas R, Zhang S, Kwon S, Sevick-Muraca EM, Li M, Chen C, Yao Q (2009). Virus-like particle (VLP) lymphatic trafficking and immune response generation after immunization by different routes. J Immunother.

[52] Zhao L, Seth A, Wibowo N, Zhao CX, Mitter N, Yu C, Middelberg AP (2014). Nanoparticle vaccines. Vaccine.

[53] Kim H, Uto T, Akagi T, Baba M, Akashi M (2010). Amphiphilic poly(amino acid) nanoparticles induce size-dependent dendritic cell maturation. Adv Funct Mater.

[54] Okuda T, Fukui A (2018). Generation of anti-oligosaccharide antibodies that recognize mammalian glycoproteins by immunization with a novel artificial glycosphingolipid. Biochem Biophys Res Commun.

[55] Vidarsson G, Dekkers G, Rispens T (2014). IgG subclasses and allotypes: from structure to effector functions. Front Immunol.

[56] Heer AK, Shamshiev A, Donda A, Uematsu S, Akira S, Kopf M, Marsland BJ (2007). TLR signaling fine-tunes anti-influenza B cell responses without regulating effector T cell responses. J Immunol.

[57] Martin RM, Brady JL, Lew AM (1998). The need for IgG2c specific antiserum when isotyping antibodies from C57BL/6 and NOD mice. J Immunol Methods.

[58] Nimmerjahn F, Ravetch JV (2005). Divergent immunoglobulin g subclass activity through selective fc receptor binding. Science.

[59] Zhou Z, Liao G, Mandal SS, Suryawanshi S, Guo Z (2015). A fully synthetic self-adjuvanting Globo H-Based vaccine elicited strong T cell-mediated Antitumor Immunity. Chem Sci.

[60] Ramirez AS, Boilevin J, Lin CW, Ha Gan B, Janser D, Aebi M, Darbre T, Reymond JL, Locher KP (2017). Chemo-enzymatic synthesis of lipid-linked GlcNAc2Man5 oligosaccharides using recombinant Alg1, Alg2 and Alg11 proteins. Glycobiology.

